# Prevalence and factors associated with hypothyroidism in children with sickle cell anemia aged 6 months − 17 years attending the Sickle Cell Clinic, Mulago Hospital, Uganda; a cross-sectional study

**DOI:** 10.1186/s12902-023-01317-2

**Published:** 2023-03-13

**Authors:** Gloria Kaudha, Thereza Piloya, Victor Musiime, Mary Goretty Kuteesa, Shamimu Namugerwa, Gloria Owomugisha, Stella Alinafe Wachepa, Sanyu Kirabo Lubwama, Sarah Kiguli, James K Tumwine

**Affiliations:** 1 Department of Paediatrics and Child Health, College of Health Sciences, Makerere University, P.O. Box 7072, Kampala, Uganda; 2 Department of Paediatrics and Child Health, Kabale University School of Medicine, Kabale, Uganda

**Keywords:** Hypothyroidism, Sickle cell anemia, Uganda, Constipation

## Abstract

**Purpose:**

Hypothyroidism has been reported at a prevalence of 6% in children and adolescents with Sickle cell anemia. In this study, we determined the prevalence and factors associated with hypothyroidism among children with Sickle cell anemia attending the Sickle Cell Clinic, in Mulago hospital, Uganda.

**Methods:**

A cross-sectional study was conducted among children aged 6 months − 17 years with a confirmed diagnosis of Sickle Cell Anemia, with no prior diagnosis of hypothyroidism and in steady state attending the Sickle Cell Clinic in Mulago hospital. Data was collected using a structured questionnaire and a blood sample was used to measure thyroid stimulating hormone and free thyroxine.

**Results:**

Of the 332 children enrolled, sixty (18.1%) participants had sub-clinical hypothyroidism (95% CI: 14.3 — 22.6). Factors associated with hypothyroidism included constipation [adjusted odds ratio: 3.1, 95% CI:1.0 — 9.0, p = 0.043] and male sex [adjusted odds ratio:2.0, 95% CI:1.1— 3.5, p = 0.025].

**Conclusion:**

Approximately 1 in 5 children (18.1%) had sub-clinical hypothyroidism. Males and children who presented with constipation were more likely to have sub-clinical hypothyroidism.

## Background

Approximately 312,000 infants are born with Sickle Cell Anaemia (SCA) globally with more than 200,000 of these found in Africa and the carrier rate is 40% in some African regions [1]. In 2006, the World Health Organization (WHO) recognized SCA as a global public health problem since it contributes to 5% of under-five deaths [2] and it is estimated that approximately 50–80% of children with sickle cell anemia die annually, before the age of 5 years [3, 4]. Within Uganda, 13.3% of the population has the sickle cell trait while about 25,000 babies are born with SCA annually and the prevalence of SCA is 0.7% [5].

Children with SCA are at risk of developing hypothyroidism [6]. The recurrent episodes of hemolysis, vaso-occlusive crises, micro-vasculature obstruction due to red blood cell entrapment, iron overload as a result of hemolysis and frequent blood transfusions in SCA are the mechanisms implicated in the development of hypothyroidism [7, 8]. This end organ damage of the thyroid gland leads to changes in the thyroid hormone levels among the children with SCA [9]. As a result of the thyroid damage, children with SCA may manifest with hypothyroidism which may result in delayed physical and sexual development [10] and other subtle signs affecting quality of life.

The prevalence of hypothyroidism among SCA patients was found, in different studies, to range between 2 and 6% [11, 12]. Ozen and others carried out a cross-sectional study in Turkey assessing 50 children and adolescents with SCA for frequency and risk factors of endocrine complications and found that the prevalence of hypothyroidism was 6% [11]. Iron overload from multiple transfusions has been postulated as a potential cause of thyroid destruction [13]. Several studies among children with SCA had autopsy reports showing significant iron deposition in the thyroid gland supporting the hypothesis that transfusion hemosiderosis and subsequent cellular damage to the thyroid gland can cause hypothyroidism [7, 8, 11, 13, 14]. Investigators also proposed that thyroid dysfunction in SCA patients may be caused by damage of thyroid tissue by vaso-occlusive crises and inflammatory mediators. Increased duration of disease with requirement of transfusion therapy of more than eight transfusions per year are predictors of iron overload [8].

Hypothyroidism may affect brain and physical development, therefore leading to growth retardation and impaired bone maturation in both mild and severe forms of SCA [10, 14].These effects are usually long term with presence of thyroid dysfunction in the form of hypothyroidism [10]. Despite the consequences of hypothyroidism in children, routine screening of children for hypothyroidism is not practiced in Uganda for even those at risk. This means that there is limited data on the prevalence of hypothyroidism in children and more so among children with SCA within the country.

Therefore, this study aimed at determining the prevalence and factors associated with hypothyroidism in children with SCA attending the Sickle Cell Clinic (SCC) in Mulago hospital in order to provide baseline information which may serve as a reference point for advocacy for routine screening and treatment of hypothyroidism in these children.

## Methods

### Study design and setting

This was a cross-sectional study to determine the prevalence and factors associated with hypothyroidism among children with sickle cell anemia aged 6 months to 17 years attending the sickle cell clinic in Mulago hospital between September and October 2020. Mulago Hospital is Uganda’s National Referral and Teaching Hospital for Makerere University. It receives patients referred from health facilities within and outside Kampala District. The SCC runs 5 days a week, has more than 15,000 registered patients, receives about 60–80 patients daily and over 70 new patients monthly. The majority (75%) of the patients are below 18 years and most are from Kampala and the surrounding districts. A third of those seen daily come in with acute illness or crises while the rest come for routine check-up and medicine refills. There is no routine screening for thyroid dysfunction at the clinic. In Uganda, our diet frequently includes goitrogens and it is common practice to use iodized salt.

### Study participants

We included children with confirmed sickle cell anemia aged 6 months to 17 years who attended the sickle cell clinic during the study period, provided assent for those 8 years and above and whose caregivers provided informed consent. We excluded children on treatment for hypothyroidism with levothyroxine or on anti-thyroid drugs such as carbimazole and propylthiouracil and those who were too ill to withstand study procedures.

### Sample size calculation

We used the formula by Scheaffer, Mendenhall III [15] to obtain the minimum sample size required to determine the prevalence of hypothyroidism in children with sickle cell anemia.

$$n = deff \times \frac{{N\widehat p\widehat q}}{{\frac{{{d^2}}}{{{{1.96}^2}}}\left( {N - 1} \right) + \widehat p\widehat q}}$$

Where.

*n* = sample size.

*deff =* Design effect = 1.

*N* = population size (N = 40 patients per day x 20 working days x 3 month study period x 75% below 18 years = 1800 ).

*p*ˆ = anticipated prevalence of hypothyroidism in children with sickle cell anemia = 50% giving the largest sample size, since there were no studies done in a similar population.

*q*ˆ = 1 - *p*ˆ = 0.5.

*d* = desired absolute precision or absolute level of precision = 5%, the study had 80% power.

***N = 317***

***Assuming 5% non-response rate adjusted sample size = 332***

### Sampling

Consecutive sampling was used; that is: every child with sickle cell anemia aged 6 months-17 years who attended the SCC during the study period and met the inclusion criteria was enrolled until the sample size was achieved.

### Study procedure

A pre-tested structured questionnaire was used to obtain the socio-demographic data, nutritional and past medical history. A detailed physical examination for signs of hypothyroidism was performed by the principal investigator or research assistant. Anthropometric measurements which included weight, height/length and mid-upper arm circumference were also recorded.

A venous blood sample of 3ml was drawn from either the ante-cubital or femoral vein using a needle and syringe and put into a plain red top vacutainer tube. This was used to measure serum thyroid stimulating hormone and free thyroxine. Fasting before the blood collection was not required. The blood samples were transported to a quality laboratory that is MBN laboratory in Kampala, within 1 h of collection. After cross-checking using the generated sample log, the samples were then centrifuged and aliquoted and analyzed on a daily basis. TSH and freeT4 levels were measured by electrochemiluminescent immunoassay technique using a fully automated COBAS 6000 ROCHE HITACHI machine from Germany. The definitions of hypothyroidism were;

TSH level > 9 mIU/L and free T4 < 0.6 ng/dL or Normal TSH and free T4 < 0.6 ng/dL were considered as clinical hypothyroidism. TSH ranging between 4.5 and10 mIU/L with normal age appropriate T4 levels that is: 6–11 months [0.9-2.0ng/dl]; 1-5years [1.0-1.8ng/dl];6–10 years [1.0-1.7ng/dl] and 11–17 years [1.0-1.6 ng/dl indicated sub-clinical hypothyroidism.

All children found with hypothyroidism were referred to the Paediatric endocrine clinic for further management.

A complete blood count (run by a SYSMEX XNL-450 machine) was also obtained from the clinic as part of their routine care and clinic protocol. A recent hemoglobin electrophoresis (within 6 months before enrollment) was also used if available to record the fetal hemoglobin levels.

### Data management

The questionnaires were numbered with serial numbers that corresponded to the labels on the blood samples. Questionnaires were checked for accuracy and completeness, and stored in a safe place under lock and key. Data was entered into the computer that is password protected using double entry method and cleaned using Epidata version 3.1 and exported to Stata version 14 for analysis.

### Data analysis

The study had 80% power, with an absolute error between the estimated and true value of 5% and with 95% confidence intervals.

Baseline characteristics were summarized as mean, standard deviation, median and interquartile range for continuous variables and frequency and proportions for categorical. The proportion of children with hypothyroidism was obtained by dividing the number of children with hypothyroidism by the number of all children with sickle cell anemia aged 6 months-17 years who were enrolled in the study. Simple logistic regression was used to test the association between hypothyroidism and independent variables individually at 5% level of significance and any variable that achieved a p < 0.2 was considered for multivariable analysis. Multiple logistic regression models were used to assess the simultaneous association between the dependent variable and independent variables. Backward elimination using an inclusion criteria p < 0.2 was used to build model of best fit. Goodness of fit test was conducted at 5% level of significance.

Interaction and confounding were assessed.

## Results

### Baseline characteristics of the study participants

We enrolled 332 children with SCA aged 6 months to 17 years between September 2020 and October 2020. The median age (IQR) was 6 [3–10] years. 40% (133 of 332) of the patients were aged less than 5 years of age. Half of the participants were female 167(50.3%). Median birth weight (IQR) was normal 3.4 (3-3.6) kg. Two (0.6%) children had a history of neck surgery (Tonsillectomy) (Table 1). As regards dietary history, most patients consumed local Ugandan diets consisting of foods known to contain goitrogens for example, 255 (76.8%) reported consuming cabbage at least once a week. Other goitrogen- rich foods consumed included cassava, 240(72.3%), sweet potatoes, 203 (61.1%), and millet, 202(60.8%).

**Table 1:** Baseline characteristics of 332 children enrolled in the study

Variable	Frequency (N = 332)	Percentage (%)
Age		
Median (IQR)	6 (3–10)	
6 months – <5 years	133	40.1
5–10 years	117	35.2
>10 years	82	24.7
Sex		
Male	165	49.7
Female	167	50.3
Nutritional status ¥		
No wasting (WFL/H and BMI z score > -2SD)	263	79.2
Wasting (WFL/H and BMI z score < -2SD)	69	20.8
Hydroxyurea		
No	106	31.9
Yes	226	68.1
Co-morbidities		
No	330	99.4
Yes	2	0.6
Birth weight n = 313		
<2.5	13	4.2
2.5–3.5	219	70
>3.5	81	25.9
Milestone achievement		
Normal	331	99.7
Delayed	1	0.3
Family History of thyroid disease		
No	314	94.6
Yes	18	5.4
Reported HIV status		
Negative	252	75.9
Positive	4	1.2
Unknown	76	22.89
Breast feeding		
No	312	94
Yes	20	6
Type of Salt		
Iodized packed salt	262	78.92
Other	70	21.08

Preterm*- A baby born before 37 completed weeks of gestation ¥WFL/H -Weight for length/height, BMI-Body mass index.

### Maternal characteristics for the enrolled participants

The median age (IQR) was 32 (27–37) years. Eighteen mothers (5.4%) reported being HIV positive. Most of the mothers reported consuming iodized packed salt 270/331 (81.6%) during pregnancy and lactation. There was no positive history of neck swellings reported by the mothers (Table 2).

**Table 2:** Maternal characteristics for the children enrolled in the study

	Frequency ( N = 332)	Percentage (%)
Age in completed years n = 330		
Median (IQR)	32 (27–37)	
20–24	31	9.4
25–29	100	30.3
30–34	78	23.6
≥ 35	121	36.7
Reported HIV status		
Negative	310	93.4
Positive	18	5.4
Unknown	4	1.2
Type of Salt n = 331		
Iodized packed salt	270	81.6
Other	61	18.4

### Sickle cell disease factors and clinical characteristics of the patients

Half the patients had not received blood transfusions in the last year and half of the patients reported having at least 2 vaso-occlusive crises (VOCs) (IQR 1–3).

Forty five out of 332 patients (13.6%) reported a history of stroke. Only 3 patients reported a history of acute chest syndrome and /or avascular necrosis. About a third of the patients, 106(31.9%) reported a history of fatigue/exercise intolerance. More than half of the patients, 208 (62.7%) reported a history of excess weight gain. Seventeen patients reported a history of constipation. No child reported a history of neck swelling in the past 1 year and none had a neck swelling on examination (Table 3).

**Table 3:** Sickle cell disease factors and clinical characteristics of the enrolled children

Variable		Median (IQR)		
Transfusions in the last 1 year		0 (0–1)		
VOCs in the last 1 year		2 (1–3)		
Hospitalizations in the last 1 year		0 (0–1)		
Day care admissions in the last 1 year		1 (0–1)		
Variable	**Frequency** **(N = 332)**		**Percentage** **(%)**	
(History of)				
Stroke				
Yes	45		13.6	
Acute chest syndrome				
Yes	3		0.9	
Leg ulcers				
Yes	13		3.9	
Avascular necrosis				
Yes	3		0.9	
Splenic Sequestration				
Yes	7		2.1	
Cold intolerance				
Yes	113		34	
Excess weight gain				
Yes	208		62.7	
Fatigue/exercise intolerance				
Yes	106		31.9	
Constipation				
Yes	17		5.1	

### Laboratory findings of the enrolled children

The mean hemoglobin concentration was 7.99$\pm$1.25 g/dl. The mean TSH was 3.01$\pm$1.75mU/L while the average Free thyroxine was 1.36±1.75 ng/dl as shown in Table 4.

**Table 4:** Laboratory findings of the 332 children enrolled in the study

Variable	Mean	SD(+/-)
Hemoglobin concentration(g/dl)	7.99	1.25
Hematocrit (%)	25.12	3.44
White cell count(10^9/L)	14.36	6.23
Absolute Neutrophil count(10^9/L)	7.06	4.50
Platelet count(10^9/L)	468.02	168.90
Level of HBF (Hb electrophoresis within last 6 months) (%)	12.66	7.46
Thyroid function tests		
TSH (mU/L)	3.01	1.75
Free thyroxine(ng/dl)	1.36	0.17

### Prevalence of hypothyroidism in the studied population

**Fig. 1:**
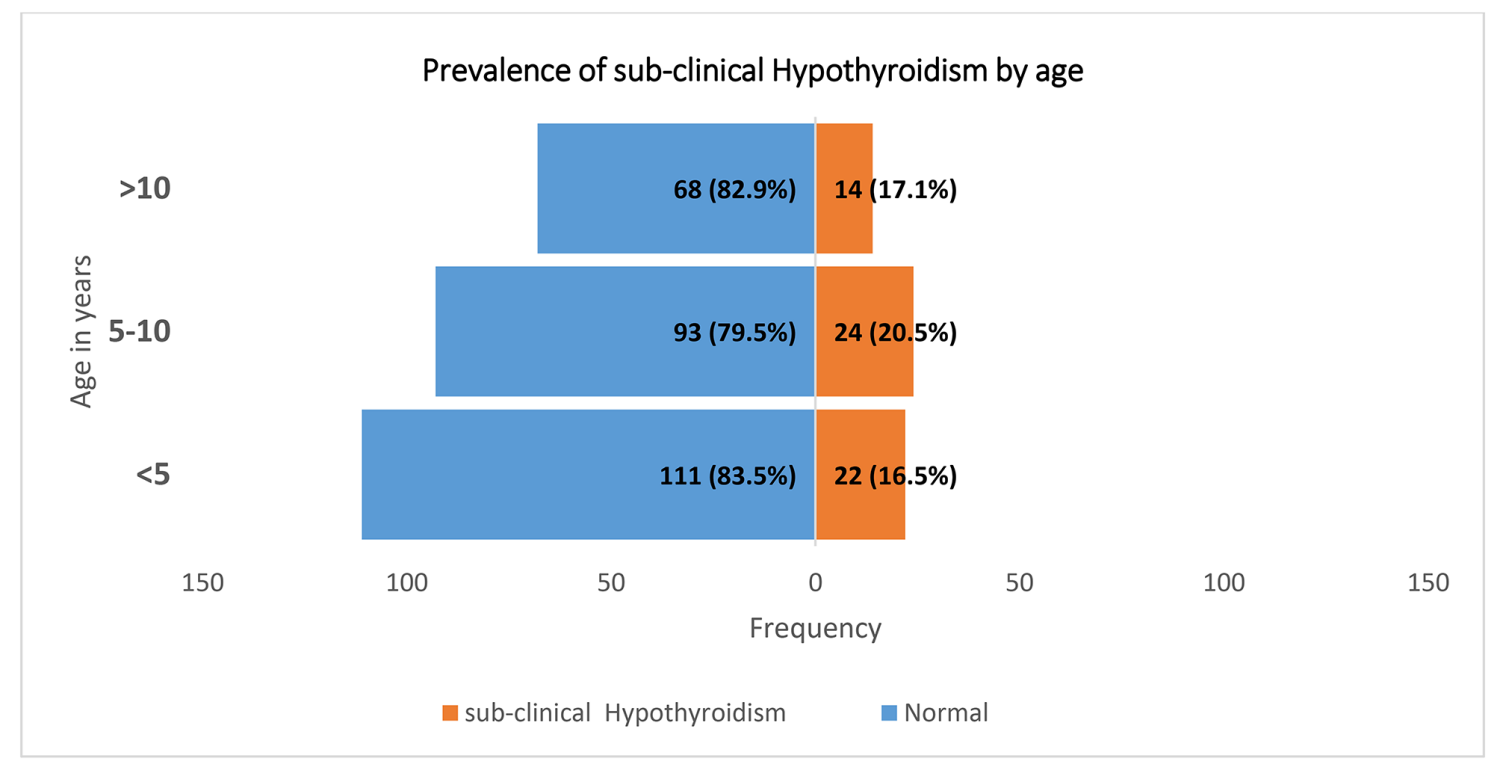
Prevalence of sub-clinical hypothyroidism by age

There was no child found to have central or primary hypothyroidism in this study, however, 18.1% [60/332 (95% CI 14.3–22.6)] of the children enrolled were found to have sub-clinical hypothyroidism (Table 5). Of those with subclinical hypothyroidism, 37/60 (61.7%) were males, 24/60 (20.5%) were between the age of 5–10 years, 22/60 (16.5%) below 5 years and 14/60 (17.1%) aged more than10 years (Fig. 1).

**Table 5:** Prevalence of hypothyroidism in the studied population

Variable	Frequency (N = 332)	Percentage (%)
Primary hypothyroidism TSH level > 9 mU/L and free T4 < 0.6 ng/dL	**0**	0
Central hypothyroidism Normal TSH/ low TSH level and free T4 < 0.6 ng/dL	0	0
Sub-clinical hypothyroidism TSH ≥ 4. 5mU/L and Normal free T4	**60**	18.1
Normal T4 / Normal TSH	**272**	81.9

### Factors associated with hypothyroidism at bivariate and multivariate analysis

At bivariable analysis, children aged 5–10 years had the highest prevalence of hypothyroidism, 24/117 (20.5%). Males had a higher prevalence of hypothyroidism 37/165 (22.4%) than females 23/167 (13.8%). The prevalence of hypothyroidism was similar among the patients who reported use of packed iodized salt and those who used other non-iodized salts 47/262 (17.9%) versus 13/70 (18.6%). The prevalence of hypothyroidism was higher among patients with a history of constipation 6/17 (35.3%) than those who did not have a history of constipation 54/315 (17.1%). The median number of hospitalizations was 1(IQR: 0 — 2) among patients with hypothyroidism compared to 0 (IQR: 0–1) among those with no hypothyroidism. The mean hemoglobin of patients with hypothyroidism, 7.75$\pm$1.3 g/dl was lower than that of patients with no hypothyroidism 8.05$\pm$1.23 g/dl (Table 6).

**Table 6:** Bivariable and Multivariable analysis showing patient factors associated with hypothyroidism

Variable	Total	Hypothyroidism			OR (95% CI)	p value	aOR (95%CI)	P value
		**No**	**Yes**					
Age category								
6 months < 5 years	**133**	111 (83.5)	22 (16.5)	1				
5–10 years	**117**	93 (79.5)	24 (20.5)	1.3 (0.69–2.47)		0.419		
>10 years	**82**	68 (82.9)	14 (17.1)	1.04 (0.50–2.17)		0.919		
Sex								
Female	**167**	**144 (86.2)**	**23 (13.8)**	**1**				
Male	**165**	**128 (77.6)**	**37 (22.4)**	**1.81 (1.02–3.21)**		**0.042**	1.96 (1.09–3.53)	**0.025**
Hydroxyurea								
No	**106**	83 (78.3)	23 (21.7)	1				
Yes	**226**	189 (83.6)	37 (16.4)	0.71 (0.40–1.26)		0.241		
Family history of thyroid disease								
No	**314**	256 (81.5)	58 (18.5)	1				
Yes	**18**	16 (88.9)	2 (11.1)	0.55 (0.12–2.47)		0.436		
Maternal HIV status								
Negative	**310**	256 (82.6)	54 (17.4)	1				
Positive	**18**	12 (66.7)	6 (33.3)	2.37 (0.85–6.59)		0.098		
Acute chest syndrome								
No	**329**	271 (82.4)	58 (17.6)	1				
Yes	**3**	1 (33.3)	2 (66.7)	9.34 (0.83-104.79)		0.07		
Constipation								
No	**315**	261 (82.9)	54 (17.1)	1				
Yes	**17**	11 (64.7)	6 (35.3)	2.64 (0.93–7.44)		0.067	3.05 (1.04–8.97)	**0.043**
Hemoglobin concentration mean (sd)		8.05 (1.23)	7.75 (1.3)	0.82 (0.65–1.03)		0.092		
Hematocrit mean (sd)		25.26 (3.45)	24.45 (3.38)	0.93 (0.86–1.01)		0.1		
Number of hospitalizations*				1.23 (0.98–1.53)		0.072	1.22 (0.97–1.53)	0.087

At multivariable analysis, male participants had 2-fold higher odds of having hypothyroidism than female counterparts (aOR:2.0, 95% CI:1.1 — 3.5, p = 0.025). Patients who reported a history of constipation had 3-fold higher odds of having hypothyroidism than patients who had no history of constipation (aOR: 3.1, 95% CI:1.0 — 9.0, p = 0.043) (Table 6.

## Discussion

This study aimed to determine the prevalence and factors associated with hypothyroidism among children with sickle cell anemia aged 6-months to 17 years in Uganda. We found that 18.1% of the children with SCA had hypothyroidism and all these had sub-clinical hypothyroidism. The prevalence of sub-clinical hypothyroidism in this study was higher in comparison to other studies that have reported a prevalence ranging from 2 to 6% [11, 13, 16]. The reason for the high prevalence of in this study could have been due to the bigger sample size as compared to the ones in the different studies For example in one of the studies, Ozen and colleagues assessed only 50 participants [11] while we assessed 332. Being a rare disease, using a smaller sample size may underestimate the prevalence. The higher prevalence may also be explained by the fact that in this study, we also considered sub-clinical hypothyroidism which has been found to have a higher prevalence as compared to clinical hypothyroidism in the general population. The goitrogen-rich Ugandan diet that most of the participants consumed in this study, could further explain the high prevalence as more than two-thirds of the children in this study consumed at least one goitrogen-rich food on a weekly basis. This was however not statistically significant and no child was found to have a neck swelling suggestive of goiter despite the high intake of goitrogen-rich foods in this study population.

However the prevalence in this study was lower than that in a case control study done in Egypt that found a prevalence of 21.7% [17]. This difference may be due to the fact that we only assessed children with SCA while the Egyptian study assessed children with sickle cell disease (SCD). The prevalence of sub-clinical hypothyroidism in this study was 18.1% which was higher than the 10% [17] reported in a case control study. This difference could be explained by the difference in the sample size (332 in the current study versus 60 in the Egyptian study) but also the difference in the population studied. Although we studied children with SCA aged 6 months to 17 years, only about a quarter (24.7%) were aged 10 years or more whereas the other population consisted of patients with SCD aged 10 years and above (adolescents) in whom sub-clinical hypothyroidism has been shown to be higher [18].

Surprisingly, our findings show that the prevalence of sub-clinical hypothyroidism was similar between children who used iodized packed salt and those who did not. One would have expected a higher prevalence among those who used non-iodized salt as it is a risk factor for hypothyroidism. However, because we were unable to objectively quantify the amount of iodine taken in by this population, we cannot ascertain if there was an actual difference between the 2 groups.

The prevalence was higher in males as compared to females (22.4% versus 13.8% respectively). This is in keeping with a study by Parshad who found that males with SCA had lower endogenous T3 hormone and high TSH levels which could put them at a higher risk for hypothyroidism as compared to their female counterparts [7].This finding is in contrast to that in a study by Ozen et al., which did not find a significant association between gender and hypothyroidism in SCA[11]. This difference may also be explained by the sample size. We used a larger sample size almost 6.5 times that used in the comparison study.

The prevalence was also higher in children aged 5–10 years (20.5%) as compared to the other age categories. Although age was not significantly associated with sub-clinical hypothyroidism, we postulate that the prevalence would be higher in children older than 10 years. This is because the mechanisms implicated in the pathophysiology of hypothyroidism in SCA, for example tissue ischemia, are more likely to damage the thyroid gland if they occur over a longer period [19]. The iron overload also postulated as a mechanism for thyroid injury [19] is as a result of recurrent transfusions as iron builds up over time in the thyroid gland, thus the older the child is, the more likely he or she is likely to get have received multiple transfusions resulting into iron overload. However, this finding is in contrast to that of Ozen et al., who also reported that age was significantly associated with hypothyroidism in SCA [11].

Half of the study population reported no history of a blood transfusion and no history of hospitalization in the last 1 year and the average hemoglobin concentration was7.99$\pm$1.25 g/dl. These findings may be due to the fact that more than two thirds of the study population was on hydroxyurea and thus more hemoglobin F and less crises. It is possible that the prevalence of sub-clinical hypothyroidism would have been even higher in our population if not for the high hydroxyurea uptake as these factors are important in the pathophysiology of hypothyroidism in SCA as reported by Soliman et al. [19]. Hydroxyurea may be protective in the prevention of hypothyroidism as it has been shown to increase hemoglobin F, thus reducing transfusion dependence and iron overload, one of the mechanisms put forward as a cause of hypothyroidism in SCA [20]. This may explain the absence of clinical/overt hypothyroidism in this study.

Regarding the symptoms, our study population reported a history of constipation, fatigue/exercise intolerance and excessive weight gain. These symptoms are common in both hypothyroidism and in sickle cell anemia as reported elsewhere [21]. However, only constipation was statistically significant as an association. Of the children found to be hypothyroid, 17.3% reported a history of excessive weight gain, however, hypothyroidism has been shown to be more of a consequence rather than a cause of the weight gain [22].

Males had 2 times higher odds of having sub-clinical hypothyroidism when compared with the females. This is most likely an incidental finding as studies have shown that sub-clinical hypothyroidism is more common in females. This finding is in contrast to that of Ozen et al. who did not find a significant association between sex and hypothyroidism in SCA [11]. The sample size in Ozen’s study was 50 compared to 332 in this study and thus this could explain the difference in this finding. However, there is paucity of data on the association of gender with sub-clinical hypothyroidism and more studies are needed to elucidate this.

Children who reported a history of constipation had 3-fold higher odds of having sub-clinical hypothyroidism compared to those who did not report a history of constipation. This is not surprising since constipation is a symptom of hypothyroidism [23], however drugs used in management of SCA for example morphine (used for management of painful crises) can cause it [21]. None the less, none of the children who reported a history of constipation was on morphine.

## Strengths and limitations of the study

This is probably the first study in Uganda to determine the prevalence and factors associated with hypothyroidism in children with SCA. The study had a large sample-size, therefore, the findings may be generalizable to children with SCA in other urban referral settings. In addition, consecutive sampling was used; therefore, sampling bias was reduced. Training of research assistants and regular calibration of study instruments was done, which eliminated information bias.

Due to the cross-sectional design of the study, we were unable to assess the causal relationship between hypothyroidism and SCA. We were also unable objectively assess the goitrogen-rich diet and iodine intake and yet these are common causes of hypothyroidism. We were also unable to ascertain the serum ferritin levels in this population due to financial constraints. Despite these limitations, given the large sample size and the detailed laboratory procedures, the study findings provide useful insights for longitudinal evaluations. Further studies to assess the causal relationship between hypothyroidism and SCA and outcomes of hypothyroidism in SCA are needed in our setting. Further studies are needed to establish the impact of hypothyroidism, especially sub-clinical hypothyroidism, in children with SCA.

## Conclusion

The prevalence of hypothyroidism in the study population was high and this was mainly sub-clinical. Being male and a history of constipation had 2 times and 3 times higher odds of having hypothyroidism respectively. Age was not significantly associated with hypothyroidism in this population. Clinicians need to screen all children with SCA especially males, and those who report a history of constipation for hypothyroidism and those found to have it treated appropriately.

## Data Availability

The original data set will be made available by the corresponding author upon reasonable request.

## List of Abbreviations

BMI: Body Mass Index
COVID: Corona Virus Disease
FT4: free thyroxine
SCA: Sickle cell anemia
SCC: Sickle cell clinic
SCD: Sickle Cell Disease
SOMREC: School of Medicine Research and Ethics Committee
TSH: Thyroid Stimulating Hormone
VOC: Vaso-occlusive crisis
WHO: World Health Organization

## References

[1] Piel FB, Patil AP, Howes RE, Nyangiri OA, Gething PW, Dewi M (2013). Global epidemiology of sickle haemoglobin in neonates: a contemporary geostatistical model-based map and population estimates. The Lancet.

[2] World Health A (2006). Sickle-cell anaemia: report by the Secretariat.

[3] Organization WH. Management of haemoglobin disorders: report of a joint WHO-TIF meeting, Nicosia, Cyprus, 16–18 November 2007. 2008.

[4] Grosse SD, Odame I, Atrash HK, Amendah DD, Piel FB, Williams TN. Sickle Cell Disease in Africa: A Neglected Cause of Early Childhood Mortality. American Journal of Preventive Medicine. 2011;41(6, Supplement 4):S398-S405. 10.1016/j.amepre.2011.09.013 PMC3708126 22099364

[5] Ndeezi G, Kiyaga C, Hernandez AG, Munube D, Howard TA, Ssewanyana I (2016). Burden of sickle cell trait and disease in the Uganda Sickle Surveillance Study (US3): a cross-sectional study. The Lancet Global health.

[6] Hagag AA, El-Asy HM, Badraia IM, Hablas NM, El-Latif AEA. Thyroid Function in Egyptian Children with Sickle Cell Anemia in Correlation with Iron Load. Endocrine, metabolic & immune disorders drug targets. 2019;19(1):46–52. 10.2174/1871530318666180912153349 30207251

[7] Parshad O, Stevens MCG, Hudson C, Rosenthal J, Melville GN, Dunn DT (1989). Abnormal thyroid hormone and thyrotropin levels in homozygous sickle cell disease. Clin Lab Haematol.

[8] Fung EB, Harmatz PR, Lee PDK, Milet M, Bellevue R, Jeng MR (2006). Increased prevalence of iron-overload associated endocrinopathy in thalassaemia versus sickle-cell disease. Br J Haematol.

[9] Garadah TS, Jaradat AA, Alalawi ME, Hassan AB (2016). Hormonal and echocardiographic abnormalities in adult patients with sickle-cell anemia in Bahrain. J blood Med.

[10] Sarici D, Akin MA, Kurtoglu S, Gunes T, Ozturk MA, Akcakus M (2012). Thyroid functions of neonates with Down syndrome. Ital J Pediatr.

[11] Ozen S, Unal S, Erçetin N, Taşdelen B (2013). Frequency and risk factors of endocrine complications in turkish children and adolescents with sickle cell anemia. Turkish J haematology: official J Turkish Soc Haematol.

[12] Rees DC, Williams TN, Gladwin MT (2010). Sickle-cell disease. Lancet (London England).

[13] Phillips G, Becker B, Keller VA, Hartman Iv J (1992). Hypothyroidism in adults with sickle cell anemia. Am J Med.

[14] Evliyaoglu N, Kilinc Y, Sargin O (1996). Thyroid functions in mild and severe forms of sickle cell anemia. Pediatr Int J.

[15] Scheaffer RL, Mendenhall W, Ott RL, Gerow KG (1990). Elementary survey sampling.

[16] El-Hazmi MAF, Bahakim HM, Al-Fawaz I (1992). Endocrine functions in Sickle Cell Anaemia Patients. J Trop Pediatr.

[17] ElAlfy MS, El-Sherif NH, Sakr HM, El Ashkar MNM (2019). Thyroid hemodynamic alterations in egyptian patients with sickle cell disease: relation to disease severity, total body iron and thyroid function. Expert Rev Hematol.

[18] Biondi B, Cooper DS (2008). The clinical significance of subclinical thyroid dysfunction. Endocr Rev.

[19] Soliman AT, De Sanctis V, Yassin M, Wagdy M, Soliman N (2017). Chronic anemia and thyroid function. Acta bio-medica: Atenei Parmensis.

[20] Zekavat OR, Makarem AR, Haghpanah S, Karamizadeh Z, Javad P, Karimi M (2014). Hypothyroidism in β-Thalassemia Intermedia patients with and without Hydroxyurea. Iran J Med Sci.

[21] Masika L, Hsieh M, Zhao Z, Yu X, Soldin S. Are we Missing Hypothyroidism in Sickle Cell Disease.Ann Clin Case Rep2017;2. 2017;1246.

[22] Gallizzi R, Crisafulli C, Aversa T, Salzano G, De Luca F, Valenzise M (2018). Subclinical hypothyroidism in children: is it always subclinical?. Ital J Pediatr.

[23] El-Shafie KT (2003). Clinical presentation of hypothyroidism. J Family Community Med.

